# Increased Pathogen Identification in Vascular Graft Infections by the Combined Use of Tissue Cultures and 16S rRNA Gene Polymerase Chain Reaction

**DOI:** 10.3389/fmed.2018.00169

**Published:** 2018-06-04

**Authors:** Evelyne Ajdler-Schaeffler, Alexandra U. Scherrer, Peter M. Keller, Alexia Anagnostopoulos, Michael Hofmann, Zoran Rancic, Annelies S. Zinkernagel, Guido V. Bloemberg, Barbara K. Hasse, and the VASGRA Cohort, A. Anagnostopoulos

**Affiliations:** ^1^Division of Infectious Diseases and Hospital Epidemiology, University Hospital and University of Zurich, Zurich, Switzerland; ^2^Institute of Medical Microbiology, University of Zurich, Zurich, Switzerland; ^3^Clinic for Cardiovascular Surgery, University Hospital and University of Zurich, Zurich, Switzerland

**Keywords:** 16S rRNA gene polymerase chain reaction, vascular graft infection, pathogen identification, microbiological diagnosis, tissue culture

## Abstract

**Background:** Vascular graft infections (VGI) are difficult to diagnose and treat, and despite redo surgery combined with antimicrobial treatment, outcomes are often poor. VGI diagnosis is based on a combination of clinical, radiological, laboratory and microbiological criteria. However, as many of the VGI patients are already under antimicrobial treatment at the time of redo surgery, microbiological identification is often difficult and bacterial cultures often remain negative rendering targeted treatment impossible. We aimed to assess the benefit of 16S rRNA gene polymerase chain reaction (broad-range PCR) for better microbiological identification in patients with VGI.

**Methods:** We prospectively analyzed the clinical, microbiological, and treatment data of patients enrolled in the observational Vascular Graft Cohort Study (VASGRA), University Hospital Zurich, Switzerland. The routine diagnostic work-up involved microbiological cultures of minced tissue samples, and the use of molecular techniques in parallel. Patient-related and microbiological data were assessed in descriptive analyses, and we calculated sensitivity, specificity, negative and positive predictive value for broad-range 16S rRNA gene PCR versus culture (considered as gold standard).

**Results:** We investigated 60 patients (median age 66 years (Interquartile range [IQR] 59–75)) with confirmed VGI between May 2013 and July 2017. The prevalence of antimicrobial pretreatment at the time of sampling was high [91%; median days of antibiotics 7 days (IQR 1–18)]. We investigated 226 microbiological specimens. Thereof, 176 (78%) were culture-negative and 50 (22%) were culture-positive. There was a concordance of 70% (158/226) between conventional culture and broad-range PCR (sensitivity 58% (95% CI 43–72); specificity 74% (67–80%)). Among the group of 176 culture-negative specimens, 46 specimens were broad-range PCR-positive resulting in identification of overall 69 species. Among the culture and/or broad-range PCR-positive specimens (*n* = 96), 74 (77%) were monomicrobial and 22 (23%) polymicrobial, whereas the rate of polymicrobial samples was higher in broad-range PCR-positive specimens (93%).

**Conclusions:** Combined cultures and broad-range 16S rRNA gene PCR from periprosthetic tissue and/or explanted vascular grafts increased the diagnostic accuracy in VGI, particularly in patients already under antimicrobial treatment at the time of redo surgery. Ideally, antimicrobial treatment should be withheld until surgical sampling in order to optimize microbiological diagnostics.Clinical trials.gov identifier: NCT01821664

## Introduction

Vascular graft infections (VGIs) are among the most serious complications in vascular surgery (1), increasing the risk of morbidity and mortality in affected patients substantially. Extracavitary groin or limb graft infections occur in about 2–6%, whereas intracavitary abdominal or thoracic graft infections are less common (0.5–2%). Vascular surgical site infections are combining the clinical features of biofilm-mediated and chronic wound infections due to both, the implantation of foreign material and circulatory disturbances. Therefore, therapy strategies solely relying on antimicrobial treatment oftentimes fail, and redo-surgery is needed. Traditional surgical salvage procedures include complete or partial graft removal with in-situ or extra-anatomical reconstruction and local treatment with muscle flap, omentum, or negative pressure wound therapy (NWPT) (1, 2).

The broadly used definition of VGI is based on clinical, radiological, and laboratory criteria (3–5). Even though histopathologic work-up is routinely recommended, no standardized cut-off levels for polymorphonuclear neutrophil counts per high power field have been defined. Microbiological evidence of pathogens remains the cornerstone of diagnosis, and the detection of microorganisms in blood cultures and/or the extraction of bacteria from the periprosthetic tissue biofilm by tissue homogenization are essential for diagnosis. However, blood cultures have a low sensitivity in detecting pathogens causing a VGI, since biofilm formation on the implant material oftentimes precludes planctonic seeding of bacteria and therefore their detection in blood (4). Cultures from the prosthetic sonicate fluid have only rarely been validated for VGIs (6), especially due to the lack of standardization caused by high rates of polymicrobial infections and different involved anatomical regions and graft materials. Broad-range 16S rRNA gene polymerase chain reaction (broad-range PCR) has not been established in the routine diagnostic work-up of VGIs yet. These molecular genetic techniques (7) are providing sensitive methods for more accurate pathogen identification, especially in case of infections with antimicrobial pretreatment and fastidious to grow or non-cultivable pathogens like *Coxiella burnetii, Tropheryma whipplei*, and Mycobacteria (8–10). The method bears the risk of sample contamination and is allegedly less effective in case of polymicrobial infections. It is estimated that up to one third of all VGI are polymicrobial, which is potentially affecting the sensitivity of broad-range PCR and therefore its effectiveness in the reliable identification of causative pathogens. We aimed to assess whether broad-range PCR in addition to conventional microbiological cultures would improve diagnostics of VGIs of patients included in the prospective, observational Vascular Graft Cohort Study (VASGRA).

## Materials and methods

### Study design and study population

All participants of VASGRA with proven VGIs between May 2013 and July 2017 were eligible for this analysis. In the VASGRA cohort clinical, laboratory and microbiological information is collected at three-monthly follow-up visits. The prospective, observational cohort is located at the University hospital of Zurich (Switzerland), a 880-bed tertiary-care referral center. We obtained written informed consent from all participants. The Independent Ethics Committee of Zurich approved the study (KEK_2012-0583).

### Definitions and classifications

We used the Management of Aortic Graft Infection Collaboration (MAGIC) criteria (5) for the diagnosis of VGI. A multidisciplinary team of infectious disease specialists, microbiologists, cardiovascular surgeons and radiologists adjudicated each criterion. In addition, each VGI was additionally classified according to the Samson classification (11). Extracavitary VGIs mainly included the groin and/or the lower extremities, whereas intracavitary VGIs involved the abdomen or less commonly the thorax (1).

### Microbiological analyses

Clinical specimens from suspected VGIs were sent to the Institute for Medical Microbiology, Zurich, Switzerland for conventional microbiological culture as well as bacterial broad-range PCR. For strict pathogens, we considered the microbiologic criterion as fulfilled when there was one positive culture and/or a positive broad-range PCR from an intraoperative specimen. For potential “contaminant” pathogens (e.g., coagulase-negative Staphylococci, *Corynebacterium* spp., *Propionibacterium acnes*), at least two positive intraoperative specimens were required.

### Microscopy

We performed Gram stains of clinical specimens according to standard procedures. Ten 1,000-fold visual fields were used to determine the mean bacterial count (no bacteria; detection of bacteria after enrichment; detection of some scattered bacteria; detection of bacteria); and ten 100-fold visual fields were used to determine the mean count for leucocytes (no leucocytes, some scattered amounts of leucocytes, few leucocytes, leucocytes in lumps).

### Cultures

Tissue specimens were placed in sterile 0.9% sodium chloride solution in the operating theater and immediately transferred to the laboratory. Upon receipt, the specimens were minced using an IKA Ultra-Turrax tissue homogenizer (Sigma-Aldrich, Buchs, Switzerland) and culture media were inoculated and incubated under aerobic and anaerobic conditions. Sheep agar plates without antibiotics (COS; bioMérieux, Marcy l'Etoile, France), and with colistin-nalidixic acid (CNA) were used for the detection of Gram-positive bacteria (bioMérieux), while MacConkey (MCK) agar plates (bioMérieux) were used for Gram-negative bacteria and Sabouraud agar plates for fungi. In addition, thioglycolate broth, aerobic selective chocolate agar plates (HAE2; bioMérieux), anaerobic sheep blood agar plates with hemin and vitamin K1 (*Brucella* agar; BD), laked sheep blood *Brucella* agar plates with kanamycin and vancomycin (BD), and phenylethylalcohol agar plates with vitamin K1 (BD) were used. Growth on agar plates was examined at 24, 48, and 72 h as well as after one week; the thioglycolate broth was examined up to day 10 and the Sabouraud agar plates up to day 20 (12). Antibiotic susceptibility testing was performed according to EUCAST recommendations (13).

### Bacterial and fungal identification

16S broad-range PCR was performed as a conventional, semi nested PCR using three amplification primers as described before (14, 15). PCR amplification was assessed optically on silver-stained polyacrylamide gel and compared to the buffer background as described by Rampini et al. (15). Reagents and buffers were routinely controlled to be low bacterial DNA-containing prior to use. Broad-range fungal inter spacer region (ITS) polymerase chain reaction (PCR) for the detection and identification of fungi in clinical specimens was performed as described before (16).

We used matrix-assisted laser desorption ionization–time of flight (MALDI-TOF) and 16S rRNA gene or ITS sequencing for identification (7, 14, 16–18). 16S rRNA sequence homology analyses were done using GenBank (NCBI) and the SmartGene IDNS database and software (SmartGene GmbH, Zug, Switzerland).

### Statistical analysis

To analyze the patient characteristics and the characteristics of the VGI, we used all available diagnostic samples (performed in internal or external laboratories). For the further analysis, we restricted the analysis to patients where i.a. deep or superficial wound tissue specimens, NPWT foams or explanted vascular grafts were investigated in parallel with broad-range PCR and conventional microbiological cultures. Samples with only broad-range PCR or microbiological cultures were excluded. We present results as medians with interquartile range (IQR), crude numbers, percentages and 95% confidence intervals (CI) of specimens/species. We compared sensitivity, specificity, positive predictive value and negative predictive value of 16S rRNA gene broad-range PCR and conventional culture (considered as gold standard) (19). We performed statistical analyses with Stata (version 15.1/SE; Stata Corporation, College Station, Texas, USA).

## Results

### Patient characteristics

**Figure 1:**
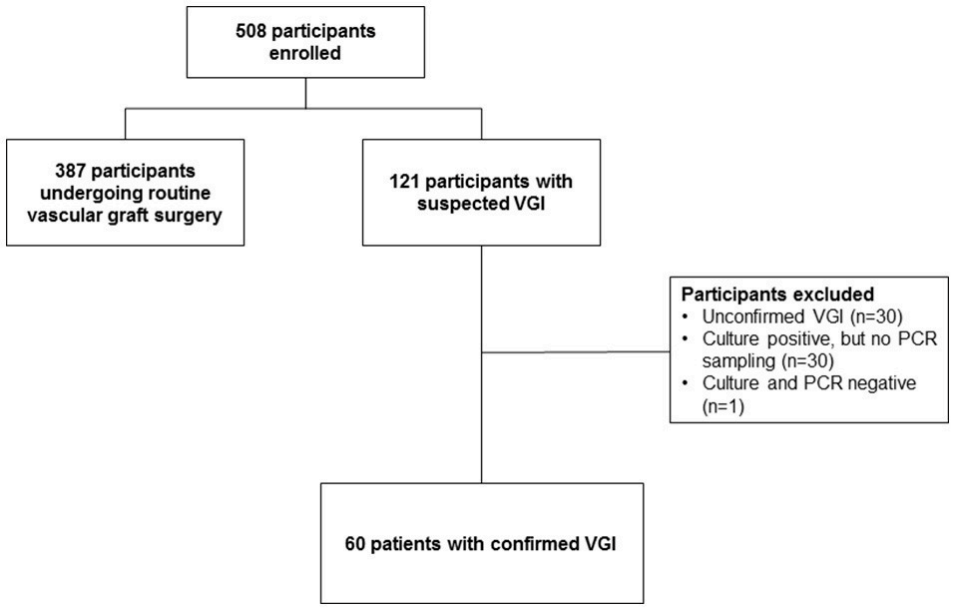
Patient selection. For this figure all microbiological samples were included (also when only broad range PCR or culture had been performed). VGI, Vascular graft infection; PCR, polymerase chain reaction.

The VASGRA dataset included information on 508 participants. We excluded 387 control patients because they underwent routine vascular surgery without infection. Of the remaining 121 patients with suspected VGI, 61 patients were excluded due to unconfirmed VGIs (*n* = 30) or due to missing parallel sampling of intraoperative tissue (*n* = 31) (Figure 1). Considering also patients with positive blood cultures (*n* = 17) we finally included 60 prospective patients with proven VGIs (82% men) in the analysis (Table 1). The median age, body mass index (BMI) and Charlson Comorbidity Index of participants were 66 years (IQR 58.5–75), 28 kg/m^2^ (25–30), and 2 (1–3), respectively. VGIs occurred after a median of 3 months (IQR 1–22) after the index surgery.

**Table 1:** Characteristics of patients and samples.

	**Patient characteristics *n* = 60**
Male *n* (%)	49 (100)
Age in years at infection, median (IQR)	66 (58–75)
Body mass index (kg/m^2^), median (IQR)	28 (25–30)
Charlson Comorbidity Index, median (IQR)	2 (1–3)
Months since index surgery, median (IQR)	3 (1–22)
Days since graft infection, median (IQR)	6 (0–22)
Localization of vascular graft	
Abdominal intracavitary, *n* (%)	33 (55)
Thoracic intracavitary, *n* (%)	11 (20)
Extracavitary, *n* (%)	13 (22)
Other, *n* (%)	3 (3)
Samson Classification	
3, *n* (%)	29 (49)
4, *n* (%)	15 (25)
5, *n* (%)	16 (26)
Specimens, *n* (%)	226 (100)
Number of specimens per patient, median (IQR)	3 (1–4)
Material, *n* (%)	
Deep wound, *n* (%)	156 (69)
Superficial wound, *n* (%)	30 (13)
Vascular graft, *n* (%)	6 (3)
Other (e.g., biopsy, blood culture), *n* (%)	20 (9)
NPWT foam, *n* (%)	14 (6)
Antibiotic treatment at sampling, *n* (%)	
Yes	206 (91)
Culture-positive on antimicrobial treatment, *n* (%)	40/206 (19)
PCR-positive on antimicrobial treatment, *n* (%)	71/206 (34)
Days on continuous antimicrobial treatment, Median (IQR)	7 (1–18)

*IQR, Interquartile range; NWPT, Negative pressure wound therapy*.

### Microbiological specimens

**Figure 2:**
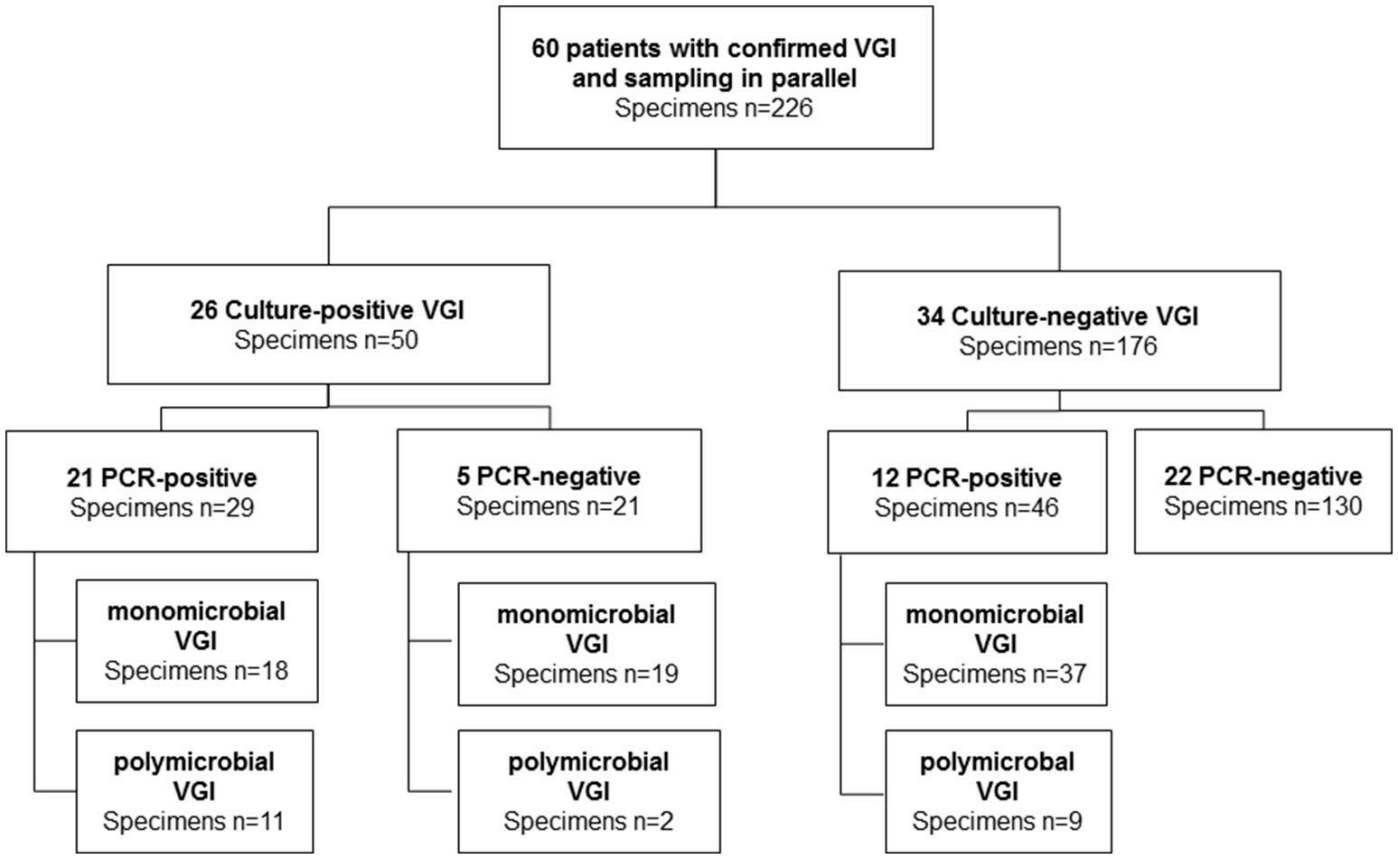
Overview of microbiological results. Here we considered only microbiological samples of patients when culture and PCR were performed in parallel. VGI, vascular graft infection; PCR, polymerase chain reaction, polymicrobial ≥2 species in one sample.

We finally investigated 226 intraoperative tissue samples in which both, tissue cultures and broad-range PCR were performed in parallel (Table 1, Figure 2). The tissue samples originated from extracavitary (22%), intracavitary abdominal (55%), or thoracic (20%) VGIs. The remaining tissue samples (3%) were isolated from secondary VGIs due to endovascular treatment of mycotic aneurysms. Microbiological samples were obtained after a median of 25 days (9-91) after diagnosis of VGI. At the time of sampling, 91% of patients were under antimicrobial treatment for a median of 7.5 days (IQR 1–18.5).

### Performance of broad-range PCR as compared to intraoperative tissue cultures

**Figure 3:**
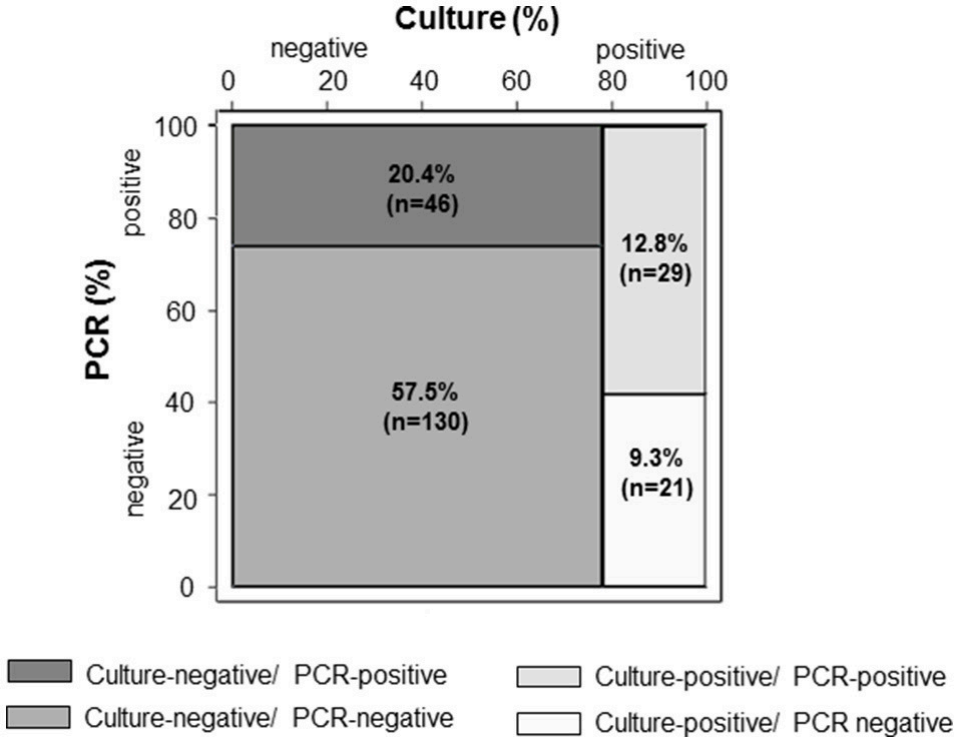
Accuracy of broad-range PCR compared to conventional culture in 226 intraoperative clinical specimens from 60 patients with confirmed vascular graft infections. PCR, polymerase chain reaction.

Among the 226 intraoperative specimens, 176 (78%) were culture-negative and 50 (22%) were culture-positive (Figures 2, 3). Among the 176 culture-negative samples, 46 were broad-range PCR-positive (≥2 organisms 20%). Among the 50 culture-positive samples (≥2 organisms 26%), 21 were broad-range PCR-negative and 29 broad-range PCR-positive (≥2 organisms 38%). Among the latter, 29 specimens, 11/29 (38%) yielded a non-identical species. Taken together, there was a concordance of 70% (159/226) between conventional culture and broad-range PCR. Compared to microbiological cultures as the gold standard, broad-range PCR had a sensitivity of 58% (95% CI 43–72), a specificity of 74% (67–80), a positive predictive value (PPV) of 39% (28–51), and a negative predictive value (NPV) of 86% (80–91). Combining culture and molecular diagnostics, the number of positive microbiological results almost doubled rising from 50/226 (22%) to 96/226 (42%). We did a sub-analysis were we ignored the presence of Candida sp, because panfungal PCR was not done for all samples. The diagnostic accuracy did not change markedly [sensitivity: 58% (39–75), specificity 76% (70–82), PPV 29% (19–42), NPV 91% (86–95)].

Among the culture and/or broad-range PCR-positive specimens (*n* = 96), 74 (77%) were monomicrobial and 22 (23%) polymicrobial. We detected in 14% two species per sample, and >3 species in 9%. The polymicrobial specimens were culture- and broad-range PCR-positive-positive in 11 cases, culture-positive and broad-range PCR-negative in two cases and culture-negative, broad-range PCR-positive-positive in nine cases.

### Detection of intraoperative species by broad-range PCR as compared to conventional cultures

The detected species in the culture and/or broad-range PCR-positive specimens were not always identical. Details on the diagnostics of the detected species are presented in Table 2 and Supplementary Table 1. Of all bacterial species, Gram-positive bacteria clearly predominated (44%) compared to Gram-negative bacteria (28%), fungi (6%), anaerobes (8%), and miscellaneous other bacteria (14%). Among all detected species, 22 (17%) were culture- and broad-range PCR-positive, 40 (30%) were culture-positive/ broad-range PCR-negative and 69 (53%) were culture-negative/broad-range PCR-positive.

**Table 2:** Overview on the detected species obtained during surgical revisions among 60 patients with confirmed vascular graft infections.

**Species in culture-positive, PCR-positive species^a^**	***n***	**(%)**	**Species in culture-positive, PCR-negative species^b^**	***n***	**(%)**	**Species in culture-negative, PCR-positive species^b^**	***n***	**(%)**
*Candida* sp.	10	(45)	*Candida* sp	8	(20)	*Staphylococcus epidermidis*	21	(30)
*Staphylococcus epidermidis*	5	(23)	*Enterococcus* spp.	6	(15)	*Streptococcus* spp.	9	(13)
*Streptococcus* spp.	2	(9)	*Propionibacterium acnes*	5	(12)	*Enterobacter* spp.	4	(6)
*Enterobacter* spp.	1	(5)	*Staphylococcus epidermidis*	5	(12)	*Corynebacterium* spp.	4	(6)
*Propionibacterium acnes*	1	(5)	*Enterobacter* spp.	2	(5)	*Klebsiella* spp.	4	(6)
*Pseudomonas aeruginosa*	1	(5)	*Eikenella corrodens*	2	(5)	*Citrobacter freundii*	3	(4)
*Streptococcus* spp.	1	(5)	*Escherichia coli*	2	(5)	*Pseudomonas aeruginosa*	3	(4)
*Mycoplasma hominis*	1	(5)	*Klebsiella* spp.	2	(5)	*Salmonella enterica*	3	(4)
			*Aspergillus fumigatus*	1	(3)	*Proteus mirabilis*	2	(3)
			*Bacteroides fragilis*	1	(3)	*Raoutella* spp.	2	(3)
			*Corynebacterium* spp.	1	(3)	*Bacteriodes xylanisolvens*	1	(1)
			*Finegoldia magna*	1	(3)	*Dialister invisus*	1	(1)
			*Mycobacterium chimaera*	1	(3)	*Escherichia coli*	1	(1)
			*Pseudomonas aeruginosa*	1	(3)	*Escherichia fergusonii*	1	(1)
			*Staphylococcus* spp.	1	(3)	*Fusobacterium nucleatum*	1	(1)
			*Streptococcus* spp.	1	(3)	*Listeria innocua*	1	(1)
						*Mycobacterium intracellulare*	1	(1)
						*Pasteurella multocida*	1	(1)
						*Pluralibacter pyrinus*	1	(1)
						*Propionibacterium acnes*	1	(1)
						*Shigella* spp.	1	(1)
						*Yokenella* spp.	1	(1)
						Polymicrobial	2	(3)
Total	22	(100)	Total	40	(100)	Total	69	(100)

a *Sample origin: vascular prosthesis (5%), deep wound cultures (80%), superficial wound culture (5%), negative pressure wound therapy foams (10%)*;

b *Sample origin: vascular prosthesis (12%), deep wound cultures (78%), superficial wound cultures (10%), biopsy (5%); ^3^Sample origin: vascular prostheses (5%), biopsy (5%), negative pressure wound therapy foams (5%), deep wound cultures (78%), superficial wound cultures (6%)*.

Culture- and broad-range PCR-positive species: Among the 22 species with positive results in both methods, *Candida* sp. (45%) and *S. epidermidis* (23%) predominated. At the time of sampling, 21/22 patients were on antimicrobial treatment for a median of 6 days (IQR 3–6).

**Figure 4:**
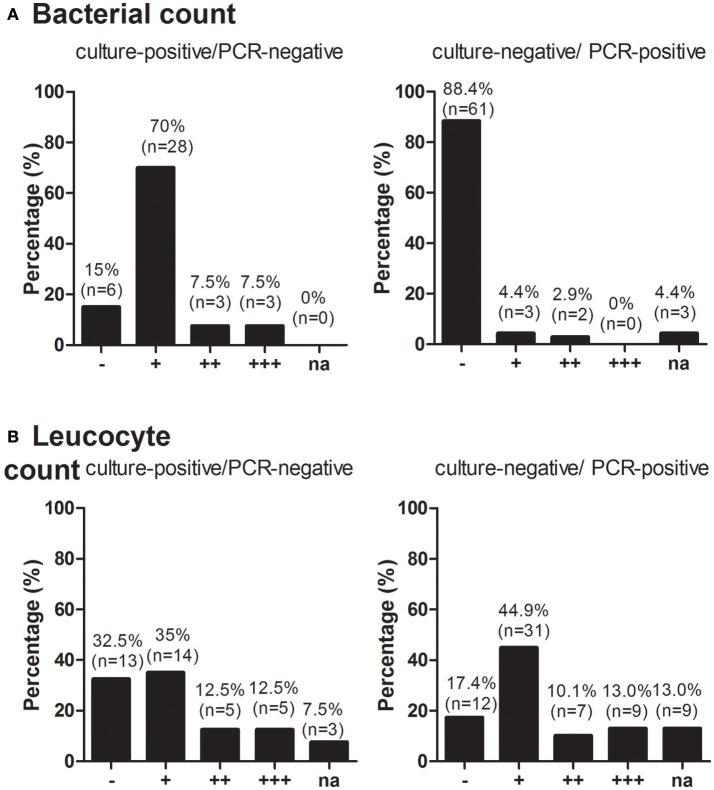
Bacterial count **(A)** and Leucocyte count **(B)** among broad-range PCR-positive/culture-negative and broad-range PCR-negative/culture-positive specimens. RNA, ribonucleic acid; PCR, polymerase chain reaction.

Culture-positive/broad-range PCR-negative species*:* The 40 species with only positive cultures, mainly included *Candida* sp. (20%), *S. epidermidis* (13%) and *Enterococcus* spp. (15%). As the bacterial load was relatively low, no bacteria were detected (or only bacteria after enrichment) in 85% (Figure 4A). Leucocytes were detected in some scattered amounts, in a few amounts or in lumps in 35, 12.5, and 12.5%, respectively (Figure 4B).

Culture-negative/broad-range PCR-positive species*:* Among the 69 broad-range PCR-positive/ culture-negative species, *S. epidermidis and Streptococcus* spp. were detected in 33%, whereas Gram-negative bacteria, anaerobes and mycobacteria were detected in 38, 4, and 7% of the remaining isolates, respectively. No bacteria per visual field were detected in 88% of these instances (Figure 4A), and no or only scattered amounts of leucocyte count were detected by visual field in 62% (Figure 4B).

## Discussion

Combining culture-based and molecular diagnostics increased the microbiological yield in a substantial proportion of patients with VGIs. Our study is the first prospective cohort study to explore the diagnostic performance of broad-range PCR for a large number of patients. Our study showed that intraoperative tissue-/explanted vascular graft sequencing combined with culture showed more sensitivity to identify the causative pathogen as compared to intraoperative tissue cultures alone. As many of the patients were under antimicrobial treatment at the time of sampling, the rate of culture-negative tissue samples was as high as 78%. In addition, potential contaminant bacterial pathogens were taken into account. The specificity (74%) of broad-range PCR was better than its sensitivity (58%).

To date, microbiological results of pre- or intraoperatively obtained cultures from different tissue samples have only rarely been assessed in VGIs (20–22). We previously analyzed the diagnostic value of bacteria cultured from NPWT-foams as compared to bacteria obtained from deep tissue perivascular samples and found that sensitivity (58%) as well as specificity (86%) from NPWT-foams were poor (20). Another study assessed the diagnostic accuracy of blood cultures in identifying the causative pathogen among 17 patients with VGIs (22). Bisharat et al. found that microorganisms in blood cultures do not necessarily indicate a causal relationship as only 17.6% of these patients had the same microorganisms in blood cultures, excised vascular grafts, or perigraft tissue cultures, respectively.

The use of molecular techniques for the diagnosis of VGI has been suggested in the past (3, 23). However, only one retrospective study has assessed the diagnostic accuracy of broad-range PCR and sonicate-fluid cultures as compared to perivascular deep tissue and/or blood cultures among 22 patients (24 cases) with VGIs (6). The study found high rates of sonicate-fluid cultures- (79%) and broad-range 16S rRNA PCR-positivity (67%). However, blood cultures, intraoperative tissue specimens and parallel PCR/deep tissue cultures were not taken routinely, therefore preventing adequate statistical analyses and exclusion of potential contaminant pathogens. In detailed analyses, we found that broad-range PCR results contributed to the diagnosis of VGI in 21/34 (62%) patients with culture-negative infections.

Comparing perivascular tissue cultures (current gold standard) and broad-range PCR, we observed a lack of sensitivity of the molecular diagnosis. Indeed, broad-range PCR was not confirmatory in molecular species identification in 40 out of 61 instances with a positive culture results. Additionally, cultures were negative in 176 cases with 46 broad-range PCR-positive samplings. We rated 14 of 69 (16%) species found in broad-range PCR-positive and culture-negative samplings as clinically irrelevant (Single detection of a “contaminant” bacterial pathogen such as *S. epidermidis, P. acnes, Corynebacterium* spp.). Low bacterial inoculum and small amounts of bacterial DNA substantiated our decision. The mismatch of 30% comparing broad-range PCR and culture is consistent with recent studies (6, 15).

We found remarkably high rates (176 specimens) of negative microbiological results among samples, obtained after preoperative therapeutic administration of antimicrobial agents. Many patients with VGIs receive antimicrobial treatment prior to hospital admission thus complicating the microbiological diagnosis. Broad-range PCR from specimens with (*n* = 206) and without antimicrobial pretreatment (*n* = 20) contributed to a microbiological diagnosis in 19 and 50% of culture-negative VGI, respectively (Supplementary Table 2). The rate of culture-negative findings was higher than in previously reported studies (6). Nevertheless, our findings extend previous reports on VGIs (6) and from studies on orthopedic infections (24, 25) that combined diagnostics with PCR and wound culture was more sensitive than deep wound cultures alone.

The high prevalence of Gram-positive cocci (*S. epidermidis, Streptococcus* spp.*, Enterococcus* spp. *and Corynebacterium* spp.) has been reported before (3, 4, 21, 26). In contrast to our expectations (6, 26, 27), we found only limited numbers of *S. aureus*, whereas Gram-negative pathogens, anaerobes and fungi were predominantly found in intracavitary abdominal or extracavitary graft infections consistent with previous reports (1).

This study has several strengths. To our knowledge, it represents the most comprehensive prospective assessment of the use of molecular microbiological methods in VGIs to date. Additionally, molecular and culture methods were obtained in parallel in order to preclude false-positive results by contaminant pathogens (23). Moreover, a multi-disciplinary team of ID specialists, cardiovascular surgeons, microbiologists, and nuclear medicine specialists adjudicated each VGIs diagnosis. Some limitations should also be noted. Molecular approaches surveying bacterial diversity provide a less biased depiction of skin microbiota than culture-based assays, but molecular approaches currently on use are unable to distinguish between 16S rRNA genes that are derived from living versus dead bacteria. Furthermore sequencing of 16S r RNA genes does not provide information regarding the gene content of plasmids. However, DNA extraction of complex microbial communities is highly influenced by the quality of the recovered DNA. Molecular techniques are cost-intensive, bear the risk of sample contamination and are allegedly less effective in case of polymicrobial infections. Ideally, samples from suspected VGIs would have been investigated as well. However, as re-operative surgery is performed only in case of confirmed infection this was not feasible.

In conclusion, in about one third of cases with culture-negative specimens, the broad-range PCR allowed a microbiological diagnosis. Culture-positive, PCR-negative isolates were present among tissue samples with either low bacterial load, fungi or in 28% of cases contaminants were detected. With regard to enhanced microbiological diagnosis of VGI, we suggest to apply deep wound cultures as well as broad-range PCR in order to increase microbiological diagnosis especially in patients already undergoing antimicrobial treatment.

## Author contributions

BH and GB designed the study. AS analyzed the data. EA-S, AS, GB, and BH wrote the first draft, and EA-S, AS, PK, AA, MH, AZ, GB, and BH wrote the final version of the manuscript. All investigators contributed to data collection and interpretation of the data, reviewed drafts of the manuscript, and approved the final manuscript.

### Conflict of interest statement

The authors declare that the research was conducted in the absence of any commercial or financial relationships that could be construed as a potential conflict of interest.

## References

[1] Wilson WR Bower TC Creager MA Amin-Hanjani S O'Gara PT Lockhart PB. Vascular graft infections, mycotic aneurysms, and endovascular infections: a scientific statement from the American Heart Association. Circulation (2016) 134:e412–60. 10.1161/CIR.0000000000000457 27737955

[2] Mayer D Hasse B Koelliker J Enzler M Veith FJ Rancic Z. Long-term results of vascular graft and artery preserving treatment with negative pressure wound therapy in Szilagyi grade III infections justify a paradigm shift. Ann Surg. (2011) 254:754–9. 10.1097/SLA.0b013e3182365864 21997817

[3] FitzGerald SF Kelly C Humphreys H Diagnosis and treatment of prosthetic aortic graft infections: confusion and inconsistency in the absence of evidence or consensus. J Antimicrob Chemother. (2005) 56:996–9. 10.1093/jac/dki382 16269550

[4] Legout L Sarraz-Bournet B D'Elia PV Devos P Pasquet A Caillaux M. Characteristics and prognosis in patients with prosthetic vascular graft infection: a prospective observational cohort study. Clin Microbiol Infect. (2012) 18:352–58. 10.1111/j.1469-0691.2011.03618.x 21883666

[5] Lyons OT Baguneid M Barwick TD Bell RE Foster N Homer-Vanniasinkam S. Diagnosis of Aortic Graft Infection: A Case Definition by the Management of Aortic Graft Infection Collaboration (MAGIC). Eur J Vasc Endovasc Surg. (2016) 52:758–63. 10.1016/j.ejvs.2016.09.007 27771318

[6] Kokosar Ulcar B Lakic N Jeverica S Pecavar B Logar M Cerar TK. Contribution of sonicate-fluid cultures and broad-range PCR to microbiological diagnosis in vascular graft infections. Infect Dis. (2017). 10.1080/23744235.2017.1418529. [Epub ahead of print]. 29260928

[7] Bosshard PP Abels S Altwegg M Bottger EC Zbinden R. Comparison of conventional and molecular methods for identification of aerobic catalase-negative gram-positive cocci in the clinical laboratory. J Clin Microbiol. (2004) 42:2065–73. 10.1128/JCM.42.5.2065-2073.2004 15131171 PMC404636

[8] Ariefdjohan MW Savaiano DA Nakatsu CH. Comparison of DNA extraction kits for PCR-DGGE analysis of human intestinal microbial communities from fecal specimens. Nutr J. (2010) 9:23. 10.1186/1475-2891-9-23 20492702 PMC2901363

[9] Dilhari A Sampath A Gunasekara C Fernando N Weerasekara D Sissons C. Evaluation of the impact of six different DNA extraction methods for the representation of the microbial community associated with human chronic wound infections using a gel-based DNA profiling method. AMB Express (2017) 7:179. 10.1186/s13568-017-0477-z 28929383 PMC5605482

[10] Oates A Bowling FL Boulton AJ McBain AJ. Molecular and culture-based assessment of the microbial diversity of diabetic chronic foot wounds and contralateral skin sites. J Clin Microbiol. (2012) 50:2263–71. 10.1128/JCM.06599-11 22553231 PMC3405613

[11] Samson RH Veith FJ Janko GS Gupta SK Scher LA. A modified classification and approach to the management of infections involving peripheral arterial prosthetic grafts. J Vasc Surg. (1988) 8:147–53. 10.1016/0741-5214(88)90402-8 3398172

[12] John AW Laboratory Procedures in Clinical Microbiology. New York, NY: Springer (2012).

[13] EUCAST EUCAST Guidelines for Detection of Resistance Mechanisms and Specific Resistances of Clinical and/or Epidemiological Importance. Version 1.0 (2013).

[14] Bosshard PP Abels S Zbinden R Bottger EC Altwegg M. Ribosomal DNA sequencing for identification of aerobic gram-positive rods in the clinical laboratory (an 18-month evaluation). J Clin Microbiol. (2003) 41:4134–40. 10.1128/JCM.41.9.4134-4140.2003 12958237 PMC193817

[15] Rampini SK Bloemberg GV Keller PM Buchler AC Dollenmaier G Speck RF. Broad-range 16S rRNA gene polymerase chain reaction for diagnosis of culture-negative bacterial infections. Clin Infect Dis. (2011) 53:1245–51. 10.1093/cid/cir692 21976460

[16] Rampini SK Zbinden A Speck RF Bloemberg GV. Similar efficacy of broad-range ITS PCR and conventional fungal culture for diagnosing fungal infections in non-immunocompromised patients. BMC Microbiol. (2016) 16:132. 10.1186/s12866-016-0752-1 27349889 PMC4924236

[17] Schulthess B Brodner K Bloemberg GV Zbinden R Bottger EC Hombach M. Identification of Gram-positive cocci by use of matrix-assisted laser desorption ionization-time of flight mass spectrometry: comparison of different preparation methods and implementation of a practical algorithm for routine diagnostics. J Clin Microbiol. (2013) 51:1834–40. 10.1128/JCM.02654-12 23554198 PMC3716085

[18] Schulthess B Bloemberg GV Zbinden R Bottger EC Hombach M. Evaluation of the bruker MALDI Biotyper for identification of Gram-positive rods: development of a diagnostic algorithm for the clinical laboratory. J Clin Microbiol. (2014) 52:1089–97. 10.1128/JCM.02399-13 24452159 PMC3993486

[19] TDR Diagnostics Evaluation Expert Panel Banoo S Bell D Bossuyt P Herring A Mabey D. Evaluation of diagnostic tests for infectious diseases: general principles. Nat Rev Microbiol. (2010) 8(12 Suppl):S17–29. 21548184

[20] Scherrer AU Bloemberg G Zbinden R Zinkernagel AS Fuchs C Frauenfelder S Prosthetic vascular graft infections: bacterial cultures from negative-pressure-wound-therapy foams do not improve diagnostics. J Clin Microbiol. (2016) 54:2190–2193. 10.1128/JCM.01102-16 27252462 PMC4963492

[21] Revest M Camou F Senneville E Caillon J Laurent F Calvet B. Medical treatment of prosthetic vascular graft infections: review of the literature and proposals of a Working Group. Int J Antimicrob Agents (2015) 46:254–65. 10.1016/j.ijantimicag.2015.04.014 26163735

[22] Bisharat N Minuhin I. Prosthetic vascular graft infections between blood and concordance of graft culture pathogen. Am J Med Sci. (2012) 344:431–35. 10.1097/MAJ.0b013e3182442eb3 22270396

[23] Taher F Assadian O Hirsch K Falkensammer J Senekowitsch C Assadian A. [Aortofemoral vascular graft infections and their prevention]. Chirurg (2015) 86:293–302. 10.1007/s00104-015-3009-x 25693780

[24] Bemer P Plouzeau C Tande D Leger J Giraudeau B Valentin AS. Evaluation of 16S rRNA gene PCR sensitivity and specificity for diagnosis of prosthetic joint infection: a prospective multicenter cross-sectional study. J Clin Microbiol. (2014) 52:3583–9. 10.1128/JCM.01459-14 25056331 PMC4187742

[25] Trampuz A Piper KE Jacobson MJ Hanssen AD Unni KK Osmon DR. Sonication of removed hip and knee prostheses for diagnosis of infection. N Engl J Med. (2007) 357:654–63. 10.1056/NEJMoa061588 17699815

[26] Hasse B Husmann L Zinkernagel A Weber R Lachat M Mayer D. Vascular graft infections. Swiss Med Wkly. (2013) 143:w13754. 10.4414/smw.2013.13754 23348860

[27] Rawson TM Lee MJ Khanna P Gopal Rao G Renton S Buckley J. Microbiological characterisation of prosthetic vascular graft infection. J Infect. (2015) 71:400–2. 10.1016/j.jinf.2015.04.008 25912614

