# Supplementary material for: Increased Pathogen Identification in Vascular Graft Infections by the Combined Use of Tissue Cultures and 16S rRNA Gene Polymerase Chain Reaction
Source: Front Med (Lausanne). 2018 Jun 4;5:169. doi: 10.3389/fmed.2018.00169 (PMC5994401; doi:10.3389/fmed.2018.00169)
Supplement: Supplementary file 1 [file Data_Sheet_1.docx]

**SUPPLEMENTARY MATERIAL**

**Supplementary Table 1- Characteristics of patients specimens in which bacterial species were only detected by 16S rRNA gene PCR**

| **Clinical specimen** | **Bacteria observed by microscopy +, ++, +++** | **Leucocytes observed by microscopy +, ++, +++** | **Bacterial species** | **Days on antibiotic therapy** | **Judged as clinically relevant** |
| --- | --- | --- | --- | --- | --- |
| Deep wound tissue | - | + | *Citrobacter koseri* | 45 | yes |
| Deep wound tissue | - | + | *Citrobacter koseri* | 50 | yes |
| Deep wound tissue | - | + | *Citrobacter koseri* | 50 | yes |
| Deep wound tissue | - | + | *Cornybacterium tuberculostearicum* | 180 | yes |
| Deep wound tissue | - | + | *Corynebacterium tuberculostearicum* | 83 | yes |
| Deep wound tissue | - | + | *Enterobacter asburiae* | 48 | yes |
| Deep wound tissue | - | + | *Enterobacter spp* | 25 | yes |
| Deep wound tissue | - | na | *Escherichia coli* | 18 | yes |
| Deep wound tissue | - | na | *Escherichia fergusonii* | 18 | no |
| Deep wound tissue | - | na | *Fusobacterium nucleatum* | 0 | no |
| Deep wound tissue | - | + | *Granulicatella adiacens* | 1 | yes |
| Deep wound tissue | - | + | *Klebsiella michiganensis* | 25 | na |
| Deep wound tissue | - | + | *Klebsiella oxytoca* | 48 | yes |
| Deep wound tissue | - | + | *Klebsiella spp* | 97 | na |
| Deep wound tissue | - | +++ | *Listeria monocytogenes* | 9 | yes |
| Deep wound tissue | - | na | *Mycobacterium Chimaera* | 1 | yes |
| Deep wound tissue | - | + | *Pluralibacter pyrinus* | 25 | yes |
| Deep wound tissue | na | +++ | *Polymicrobial* | 2 | no |
| Deep wound tissue | na | ++ | *Polymicrobial* | 2 | no |
| Deep wound tissue | - | ++ | *Proteus mirabilis* | 20 | yes |
| Deep wound tissue | - | + | *Proteus mirabilis* | 12 | yes |
| Deep wound tissue | - | + | *Pseudomonas aeruginosa* | 59 | yes |
| Deep wound tissue | - | + | *Pseudomonas aeruginosa* | 86 | yes |
| Deep wound tissue | + | +++ | *Raoultella planticola* | 0 | yes |
| Deep wound tissue | - | + | *Salmonella enterica* | 50 | yes |
| Deep wound tissue | - | + | *Salmonella enterica* | 45 | yes |
| Deep wound tissue | - | + | *Salmonella enterica* | 50 | yes |
| Deep wound tissue | - | na | *Shigella flexneri* | 18 | no |
| Deep wound tissue | - | + | *Staphylococcus epidermidis* | 11 | yes |
| Deep wound tissue | - | - | *Staphylococcus epidermidis* | 180 | yes |
| Deep wound tissue | - | + | *Staphylococcus epidermidis* | 284 | yes |
| Deep wound tissue | - | ++ | *Staphylococcus epidermidis* | 274 | yes |
| Deep wound tissue | + | +++ | *Staphylococcus epidermidis* | 5 | yes |
| Deep wound tissue | - | - | *Staphylococcus epidermidis* | 25 | yes |
| Deep wound tissue | - | +++ | *Staphylococcus epidermidis* | 8 | yes |
| Deep wound tissue | - | + | *Staphylococcus epidermidis* | 25 | yes |
| Deep wound tissue | - | + | *Staphylococcus epidermidis* | 20 | yes |
| Deep wound tissue | - | na | *Staphylococcus epidermidis* | 284 | yes |
| Deep wound tissue | - | - | *Staphylococcus epidermidis* | 15 | yes |
| Deep wound tissue | - | + | *Staphylococcus epidermidis* | 10 | yes |
| Deep wound tissue | - | - | *Staphylococcus epidermis* | 175 | yes |
| Deep wound tissue | - | - | *Staphylococcus lugdunensis* | 6 | yes |
| Deep wound tissue | - | +++ | *Staphylococcus spp* | 9 | yes |
| Deep wound tissue | + | +++ | *Staphylococcus spp* | 7 | yes |
| Deep wound tissue | - | ++ | *Staphylococcus spp* | 9 | yes |
| Deep wound tissue | - | +++ | *Staphylococcus spp* | 9 | yes |
| Deep wound tissue | - | +++ | *Staphylococcus spp* | 9 | yes |
| Deep wound tissue | - | ++ | *Streptococcus agalactiae* | 16 | yes |
| Deep wound tissue | - | ++ | *Streptococcus dysgalactiae* | 125 | yes |
| Deep wound tissue | - | + | *Streptococcus dysgalactiae* | 170 | yes |
| Deep wound tissue | - | + | *Streptococcus pyogenes* | 510 | yes |
| Deep wound tissue | ++ | + | *Streptococcus species* | 6 | yes |
| Deep wound tissue | ++ | + | *Streptococcus species* | 6 | yes |
| Deep wound tissue | - | + | *Yokenella spp* | 48 | no |
| Superficial wound | - | - | *Corynebacterium durum* | 0 | no |
| Superficial wound | - | + | *Enterobacter aerogenes* | 18 | no |
| Superficial wound | - | + | *Raoultella ornithinolytica* | 18 | no |
| Vascular graft | - | - | *Dialister invisus* | 19 | no |
| Vascular graft | - | - | *Enterobacter asburiae* | 48 | yes |
| Vascular graft | - | - | *Klebsiella oxytoca* | 48 | yes |
| Vascular graft | - | na | *Pseudomonas aeruginosa* | 69 | yes |
| Biopsy | - | na | *Corynebacterium ureicelerivorans* | 0 | no |
| Biopsy | - | - | *Pasteurella multocida* | 9 | yes |
| Biopsy |  |  | *Propionibacterim acnes* |  | na |
| Biopsy | - | ++ | *Streptococcus dysgalactiae* | 1 | yes |
| NPWT-Foams | - | - | *Bacteroides xylanisolvens* | 10 | yes |
| NPWT-Foams | - | - | *Staphylococcus epidermidis* | 11 | yes |
| NPWT-Foams | - | + | *Staphylococcus epidermidis* | 20 | yes |
| NPWT-Foams | - | na | *Streptococcus dysgalactiae* | 165 | yes |

**Abbreviations:** Negative pressure wound therapy, NPWT; Polymerase chain reaction, PCR

**Supplementary Table 2 Samples retrieved under antimicrobial therapy.**

|  | **Culture negative**  n (%) | **Culture positive**  n (%) | **Total**  n (%) |
| --- | --- | --- | --- |
| **PCR negative**, n (%) | 122 (59) | 13 (6.3) | 135 (66) |
| **PCR positive**, n (%) | 44 (21) | 27 (13) | 71 (34) |
| **Total**, n (%) | 176 (100) | 50 (100) | 206 (100) |

Abbrevations: n, number; PCR, polymerase chain reaction; RNA, ribonucleic acid
